# Complete Genome Sequence of a Mosaic Bacteriophage, Waukesha92

**DOI:** 10.1128/genomeA.00339-14

**Published:** 2014-08-21

**Authors:** A. Brooke Sauder, Brandon Carter, Christophe Langouet Astrie, Louise Temple

**Affiliations:** Department of Integrated Sciences and Technology, James Madison University, Harrisonburg, Virginia, USA

## Abstract

In this study, we determined the complete genome sequence of a mosaic bacteriophage, Waukesha92, which was isolated from soil using *Bacillus thuringiensis* as the host organism. This temperate *Myoviridae* bacteriophage has similarities to phages SpaA1 and BceA1 and the *Bacillus thuringiensis* plasmid pBMB165.

## GENOME ANNOUNCEMENT

Waukesha92 is a *Myoviridae* bacteriophage isolated from *Bacillus thuringiensis* Kurstaki, an aerobic Gram-positive endospore-forming rod belonging to the *Bacillus cereus* group (1). *B. thuringiensis* produces a protein that is used as an insecticide (2); therefore, this phage might be useful in bioengineering the bacteria to adjust the effectiveness of the toxic protein. Waukesha92 was isolated through enrichment from compost soil in Glenn Allen, Virginia (GPS coordinates 37°39′31.6″N 77°31′14.0″W).

Sequencing was done at the Genomic Sequencing and Analysis Facility at Texas A&M using an Illumina MiSeq 250-bp paired-end run with a 550-bp insert library. Reads (200,000) were assembled using 454 Newbler version 2.9 (3) into a single contig that was viewed using Consed version 25.0 (4). The average depth of coverage was 27.21×, and no areas of poor coverage were noted. The genome is likely circularly permuted, as indicated by the lack of high-coverage regions or sticky ends and the similarity to headful packaging phages.

Waukesha92 contains a linear 45,648-bp double-stranded DNA (dsDNA) genome with a G+C content of 35.45%. The genome was autoannotated using GeneMark.hmm-P version 2.8 (5, 6) and Glimmer (7), and then refined using DNAmaster (http://cobamide2.bio.pitt.edu/computer.htm). Genome annotations were later checked using RAST (8) and Artemis (9). The genome encodes 72 proteins, 22 of which were assigned a function. The genome was scanned for tRNAs using tRNAscan-se (10). Waukesha92 is predicted to be a temperate phage due to the presence of proteins characteristic of lysogenic phages, including an integrase and multiple repressors.

BLASTn (11) was used to determine that 58% of the genome of Waukesha92 has a 98% nucleotide similarity to phages SpaA1 and BceA1. These phages are known to be mosaic, containing elements of *Staphylococcus* phage MZTP02 and elements of a *Bacillus* phage allowing them to infect both hosts (12). It is hypothesized that Waukesha92 can also infect both hosts, as indicated by conservation of attachment and lysis proteins. Additionally, many structural proteins are conserved. Again using BLASTn, we determined that 45% of the genome of Waukesha92 has 91% similarity to the *Bacillus thuringiensis* plasmid pBMB165,which contains an inducible prophage (13). Waukesha92 may have acquired a piece of this element during an infection of *Bacillus thuringiensis*.

The genomes of SpaA1, BceA1, MZTP02, pBMB165, and Waukehsa92 were aligned and visualized using Mauve (14). The first 5 kb of the genome of Waukesha92 is similar to the last 5 kb of SpaA1 and the first 5 kb of pBMB165. The next 20 kb of the Waukesha92’s genome is similar to pBMB165 but is interspersed with five approximately 1-kb regions unique to Waukesha92. Furthermore, Waukesha92 has a unique 1-kb spacer after this 20-kb region. The next 15 kb of Waukesha92’s genome is similar to phages MZTP02, SpaA1, and BceA1. The final 5 kb is present in the beginning of the genome of SpaA1 and BceA1. SpaA1 and BceA1 contain the same unique 14-kb section in the middle of their genomes. Despite some variation, Waukesha92 is clearly related to SpaA1 and BceA1 (12).

### Nucleotide sequence accession number.

The complete genome of the bacteriophage Waukesha92 is deposited in GenBank, accession number KJ920400.
